# A population‐based study of palliative rectal cancer patients with an unremoved primary tumour: Symptoms, complications and management

**DOI:** 10.1111/codi.70104

**Published:** 2025-04-23

**Authors:** Gustav Kejving, Gustav Sandén, Ingrid Ljuslinder, Jörgen Rutegård, Petrus Vinnars, Martin Rutegård

**Affiliations:** ^1^ Department of Diagnostics and Intervention, Surgery Umeå University Umeå Sweden; ^2^ Department of Diagnostics and Intervention, Oncology Umeå University Umeå Sweden

**Keywords:** chemotherapy, complications, palliation, perforation, radiotherapy, rectal cancer, stoma, surgery

## Abstract

**Aim:**

Palliative rectal cancer patients typically retain their primary tumour, as trials have concluded no survival benefit of tumour resection in non‐curative patients. This patient group is understudied regarding the natural course of the remaining tumour, particularly concerning the need of surgical management.

**Method:**

This was a retrospective study on rectal cancer patients diagnosed between 2007 and 2020 in Region Västerbotten, Sweden. Data were obtained from the Swedish Colorectal Cancer Registry and chart review. Patients were excluded if treated with curative intent, underwent primary tumour resection, had a synchronous colorectal cancer, had locally recurrent colorectal cancer, or refused treatment. Patients were followed from diagnosis until death or end of follow‐up. Indications for palliative treatment, tumour‐related complications and surgical and oncological management were investigated, with a stratified analysis for study period and patient age.

**Results:**

Some 156 patients remained after applying exclusion criteria. The majority had metastasized and incurable disease (76%). Almost half suffered local complications (44%) and 48% underwent surgical intervention, due to the unremoved primary tumour. Tumour perforation occurred in 7% with a significantly higher risk in patients aged ≤75 years (*p* = 0.009). Bowel obstruction afflicted 23%, while 40% underwent stoma diversion. Almost half received chemotherapy (48%) and radiotherapy (42%), respectively.

**Conclusion:**

Rectal cancer patients with an unremoved primary tumour face a substantial risk of local complications, often necessitating surgical intervention. Therefore, the benefits of surgical resection should be carefully considered, especially for patients with a longer estimated survival. Further research is needed to accurately identify patients where tumour removal might be beneficial.

**What does this add to the literature?**

Palliative rectal cancer patients with an unremoved primary tumour are an understudied group regarding the natural progress of the disease. In this population‐based study, complications such as bowel obstruction and even tumour perforation were not rare. Palliative resection in selected patients might therefore still be considered.

## INTRODUCTION

The majority of rectal cancer patients are amenable to cure, where treatment options mainly consist of tumour resection but may also include radiotherapy, chemotherapy and targeted therapy [1]. For patients with metastasized rectal cancer, complete surgical resection of all metastatic disease may not be possible. For appropriate patients, chemotherapy and radiotherapy with a palliative intent is offered instead, while best supportive care is reserved for those deemed unfit for such treatment. In the event of mechanical obstruction caused by the primary tumour, surgical decompression, with endoluminal stenting or diverting stoma, is the usual treatment for palliative patients.

The proximity to other pelvic organs sometimes leads to invasion of, for example, the bladder, vagina, bone or pelvic nerve roots, causing numerous local issues including pain, fistulation and infection. Faecal diversion can sometimes provide relief, but pelvic pain related to rectal cancer is notoriously hard to treat [1]. Removal of the primary tumour could potentially decrease the risk of local complications from an advanced tumour, although with the added risk of postoperative complications. Such resections used to be common even in incurable patients but have fallen out of favour as accumulating evidence demonstrates a lack of survival benefit with palliative resections [2, 3]. However, this paradigm shift might have led to debilitating local symptoms for some patients, especially in those with a longer anticipated survival.

This population‐based study aimed to illustrate the natural history of rectal cancer, in particular consequences of local progression, in patients not eligible for curative treatment, during a time when palliative resections were generally not performed.

## METHODS

### Study design

Included in this study were primary rectal cancer patients diagnosed in Region Västerbotten, Sweden, a healthcare region with approximately 260 000 inhabitants, between 2007 and 2020, using the Swedish Colorectal Cancer Registry (SCRCR) [4]. Rectal cancer was diagnosed at three hospitals in this region, and most resections during the study period were performed at the Umeå University hospital. Exclusion criteria were comprised of recurrent colorectal cancer, synchronous colorectal cancer, patient refusal of curative treatment, tumour resection of the rectum with palliative intent, relocation to another healthcare region, or transition from palliative to curative intent of treatment. Patients initially treated with curative intent were included if they later transitioned to palliative care for a meaningful period, measured in months, indicating a deliberate adjustment in their rectal cancer treatment. In contrast, curative patients who suffered an acute medical event leading to their death were excluded, as their shift to palliative care reflected the severity of the event rather than an effort to optimize their rectal cancer treatment. Included patients were reviewed until time of death or end of follow‐up at 15 November 2023.

#### Registry data

The SCRCR is a national registry established in 1995, comprising all hospitals that diagnose rectal cancer in Sweden [5]. Rectal cancer is defined here as an adenocarcinoma of the large bowel within 15 cm of the anal verge, as measured by rigid sigmoidoscopy. Average completeness of 99% and overall agreement between registry and reabstracted variables at 90% for the period 2008–2015 have previously been demonstrated [4]. The SCRCR was used to collect information on age, sex, date of diagnosis, tumour staging, date of any surgery for the primary tumour, and mortality.

#### Chart review data

Chart review was performed to ascertain data on patient comorbidity, reason for palliative treatment, local complications, surgical interventions, as well as use of palliative chemo‐ and radiotherapy. Extent of disease and symptom states were determined by the first author, using all available medical records in primary and secondary care. Comorbidity was defined as the presence of a simultaneous and potentially serious disease or medical condition separate from rectal cancer. Comorbidities were categorized into the following groups: heart disease, lung disease, kidney disease, diabetes, neurological disease, rheumatic disease, venous thrombotic disease and dementia (Table S1 for definitions).

Local complications comprised those necessitating hospitalization and/or that were presumed to be related to the primary tumour. Such complications included severe haematochezia resulting in blood transfusion, bowel obstruction, tumour perforation and local pain. Bowel obstruction was categorised as either complete or partial (symptomatic while still allowing some form of faecal passage). Local pain was divided into three categories: (i) localized pain of a considerable degree, presumably caused by the primary tumour's direct overgrowth into surrounding tissue; (ii) diffuse abdominal pain, presumably related to the rectal cancer but challenging to distinguish between direct overgrowth and metastasis‐related pain and (iii) diffuse extra‐abdominal pain, presumably related to the rectal cancer, where metastasis‐related pain is more probable than pain originating from the primary tumour.

For patients receiving chemotherapy, the regimen noted was the one initially started. Surgical procedures included all operative interventions, whether directly caused by the primary tumour or not. The indication for faecal diversion was subdivided into absolute, relative, or prophylactic. Absolute denoted either symptoms of mechanical obstruction and no faecal passage and/or signs of tumour perforation. Relative meant that symptoms of mechanical obstruction were present, along with some faecal passage and/or symptoms believed to be relieved by a diverting stoma, such as pain and/or bleeding. Prophylactic denoted that no primary tumour‐related symptoms were present, but the surgeon deemed it reasonable to defunction the patient because of anticipated primary tumour‐related symptoms in the future.

Postoperative complications were defined as deviations from the usual postoperative course within 90 days of surgery, using the Clavien‐Dindo classification of surgical complications [6]. The rationale for initiating palliative care was noted, as certain patients might be classified as palliative despite the cancer being potentially oncologically resectable.

### Statistical analysis

For the descriptive analysis, frequency tables were constructed with percentages for categorical variables and median with interquartile range for continuous variables. Stratification by time (2007–2013 vs. 2014–2020) and median age of diagnosis (≤75 years vs. >75 years), respectively, was performed. When evaluating the risk of tumour perforation in the subgroup with incurable disease as indication for palliative care, age was used as a continuous variable. Group differences were analysed with the *t*‐test for continuous variables, while Fisher's exact test was used for categorical variables. Survival differences were evaluated using the log‐rank test and illustrated using Kaplan–Meier curves. Median survival estimates were calculated with 95% confidence interval (CIs). All analyses were conducted using Stata version 18.0 (StataCorp, College Station, Texas, USA).

## RESULTS

### Patients

**FIGURE 1:**
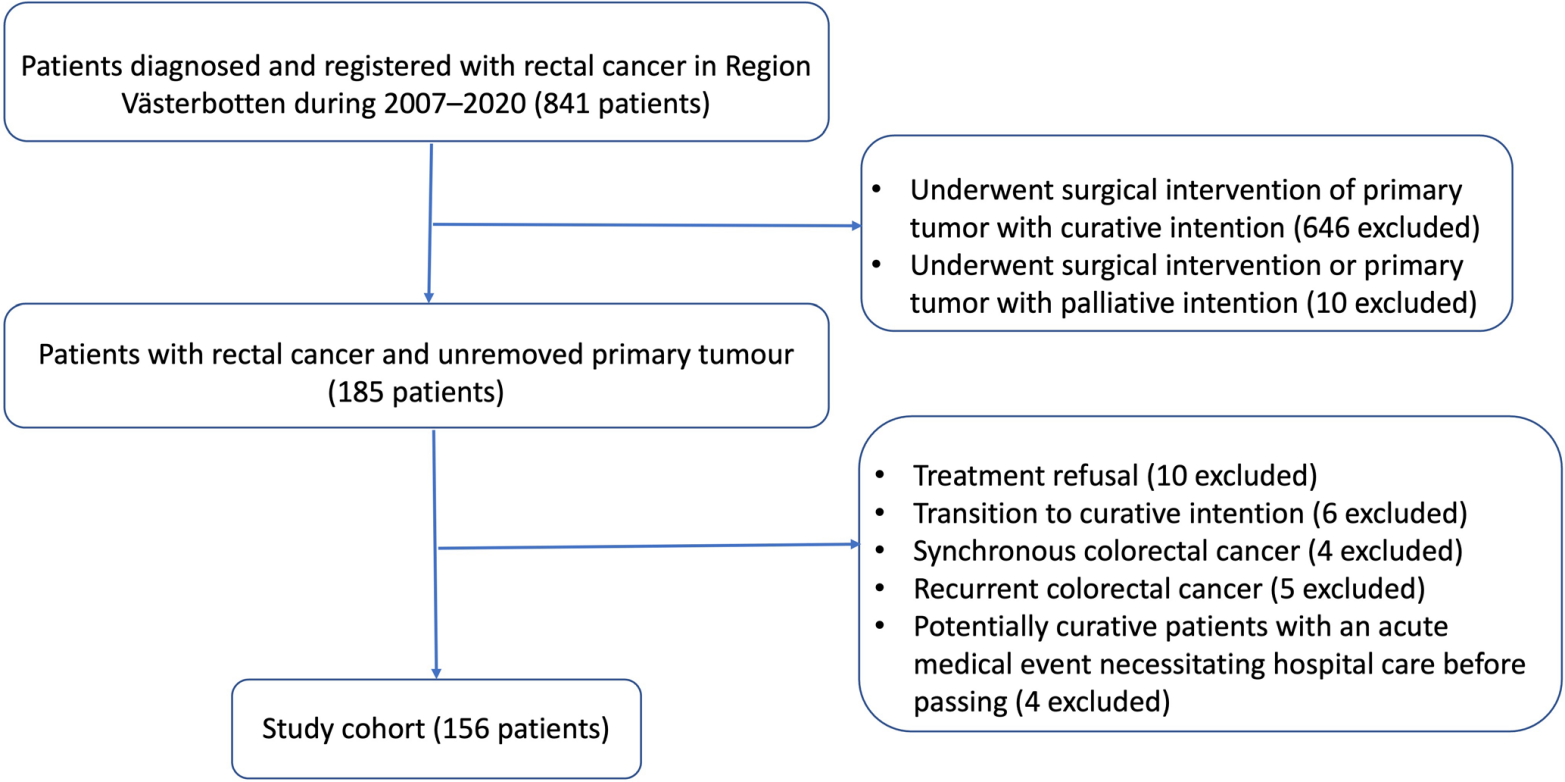
Study flow chart.

**FIGURE 2:**
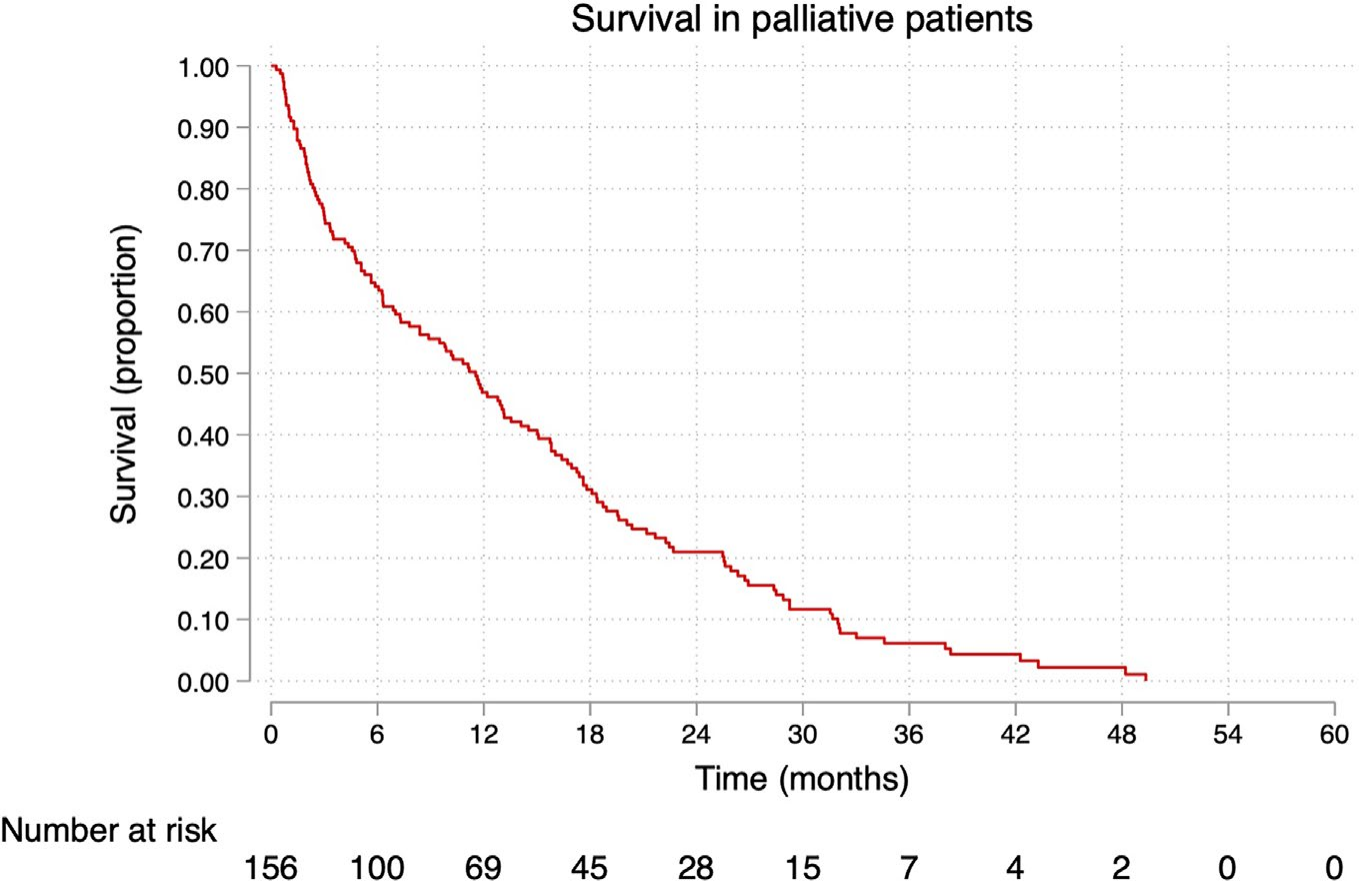
Survival in patients receiving palliative care for rectal cancer in Region Västerbotten, Sweden, diagnosed during 2007–2020.

Some 841 patients in Region Västerbotten were identified in the Swedish Colorectal Cancer Registry. Of these, 656 had a curative rectal cancer resection, while 10 patients had a palliative resection. Of the remainder, 29 patients were excluded due to synchronous colorectal cancer, recurrent colorectal cancer, treatment refusal, transition from palliative to curative treatment, or dying of an acute medical event before being considered incurable. The final study cohort consisted of 156 eligible patients (Figure 1) with a median survival of 11.5 months (95% CI: 7.8–13.5 months), as depicted in Figure 2.

The patient characteristics are listed in Table 1. The most common reason for palliative care was incurable disease due to widespread metastases. Most patients did not experience local complications nor underwent any surgical procedures related to the primary tumour. Palliative chemotherapy was slightly more common than radiotherapy. The initial oncological regimen (chemotherapy or targeted therapy) is demonstrated in Table S2.

**TABLE 1:** Baseline characteristics of 156 palliative rectal cancer patients in Region Västerbotten, Sweden, during 2007–2020.

	Total (*N* = 150)
Age (years)	75.0 (64.5–83.5)
Sex	
Male	90 (57.7%)
Female	66 (42.3%)
Year of diagnosis	
2007–2013	74 (47.4%)
2014–2020	82 (52.6%)
Number of comorbidities	
None	73 (46.8%)
One	44 (28.2%)
Two or more	39 (25.0%)
Clinical tumour stage (TNM)	
I	7 (4.5%)
II	10 (6.4%)
III	13 (8.3%)
IV	117 (75.0%)
Missing	9 (5.8%)
Main cause of palliation	
Incurable disease	119 (76.3%)
Age/comorbidity	30 (19.2%)
Staging not possible	6 (3.8%)
Other	1 (0.6%)
Local complication related to primary tumour	
No	87 (55.8%)
Yes	69 (44.2%)
Palliative radiotherapy	
No	90 (57.7%)
Yes	66 (42.3%)
Palliative chemotherapy	
No	81 (51.9%)
Yes	75 (48.1%)

*Note*: Continuous variables are presented with median (interquartile range), while categorical variables are presented with n (column %).

Abbreviation: TNM, tumour‐node‐metastasis staging system.

### Outcomes

Patient outcomes are listed in Table 2. Pain was a common symptom but relatively few (16/156, 10%) suffered from intense local pain in the pelvic area. A severe and less common primary tumour‐related event was tumour perforation (11/156, 7%). Two patients were found to have perforated rectal cancer at diagnosis, leaving nine potentially preventable tumour perforations. Median time to perforation in these patients was 366 days (range: 56–786 days): four patients had perforations before faecal diversion, while five patients perforated despite having had a diverting stoma. Complete bowel obstruction (12/156, 8%) was also uncommon. Severe haematochezia was similarly uncommon (16/156, 10%), often treated with radiotherapy (14/16, 88%). Almost half of the patients underwent surgical intervention due to the primary tumour (75/156, 48%), and more than a third underwent defunctioning stoma surgery (62/156, 40%). Four local abscess drainage and three nephrostomy procedures were performed, respectively. Debulking surgery was undertaken for three patients, which included two bilateral salpingo‐oophorectomies and one left hemicolectomy. Colonic stent due to impending bowel obstruction was performed in three patients. Other interventions included one instance of argon‐plasma coagulation treatment for suspected recurrence after radiotherapy and one osteosynthesis for a peritrochanteric femur fracture. Postoperative complications necessitating reoperative surgery (5/75, 3%) and postoperative mortality (5/75, 3%) were rare.

**TABLE 2:** Outcomes in 156 palliative rectal cancer patients in Region Västerbotten, Sweden, during 2007–2020.

	Total (*N* = 156)
Severe haematochezia	
No	140 (89.7%)
Yes	16 (10.3%)
Pain	
No pain reported	110 (70.5%)
Diffuse extra‐abdominal pain	9 (5.8%)
Diffuse abdominal pain	21 (13.5%)
Localized pain due to primary tumour	16 (10.3%)
Tumour perforation	
No	145 (92.9%)
Yes	11 (7.1%)
Degree of bowel obstruction	
None	120 (76.9%)
Partial	12 (7.7%)
Complete	24 (15.4%)
Type of stoma	
No stoma	94 (60.3%)
Ileostomy	12 (7.7%)
Colostomy	46 (29.5%)
Other	4 (2.6%)
Reason for stoma	
No stoma	94 (60.3%)
Obstruction/perforation: absolute indication	16 (10.3%)
Obstruction/pain/bleeding: relative indication	36 (23.1%)
Prophylactic	10 (6.4%)
Surgical intervention	
No	81 (51.9%)
Yes	75 (48.1%)
Postoperative complications (Clavien‐Dindo grade) within 90 days	
0–I	51 (68.9%)
II	9 (12.2%)
IIIa	4 (5.4%)
IIIb	5 (6.8%)
IV	0 (0%)
V	5 (6.8%)

### Stratification by year of diagnosis

**FIGURE 3:**
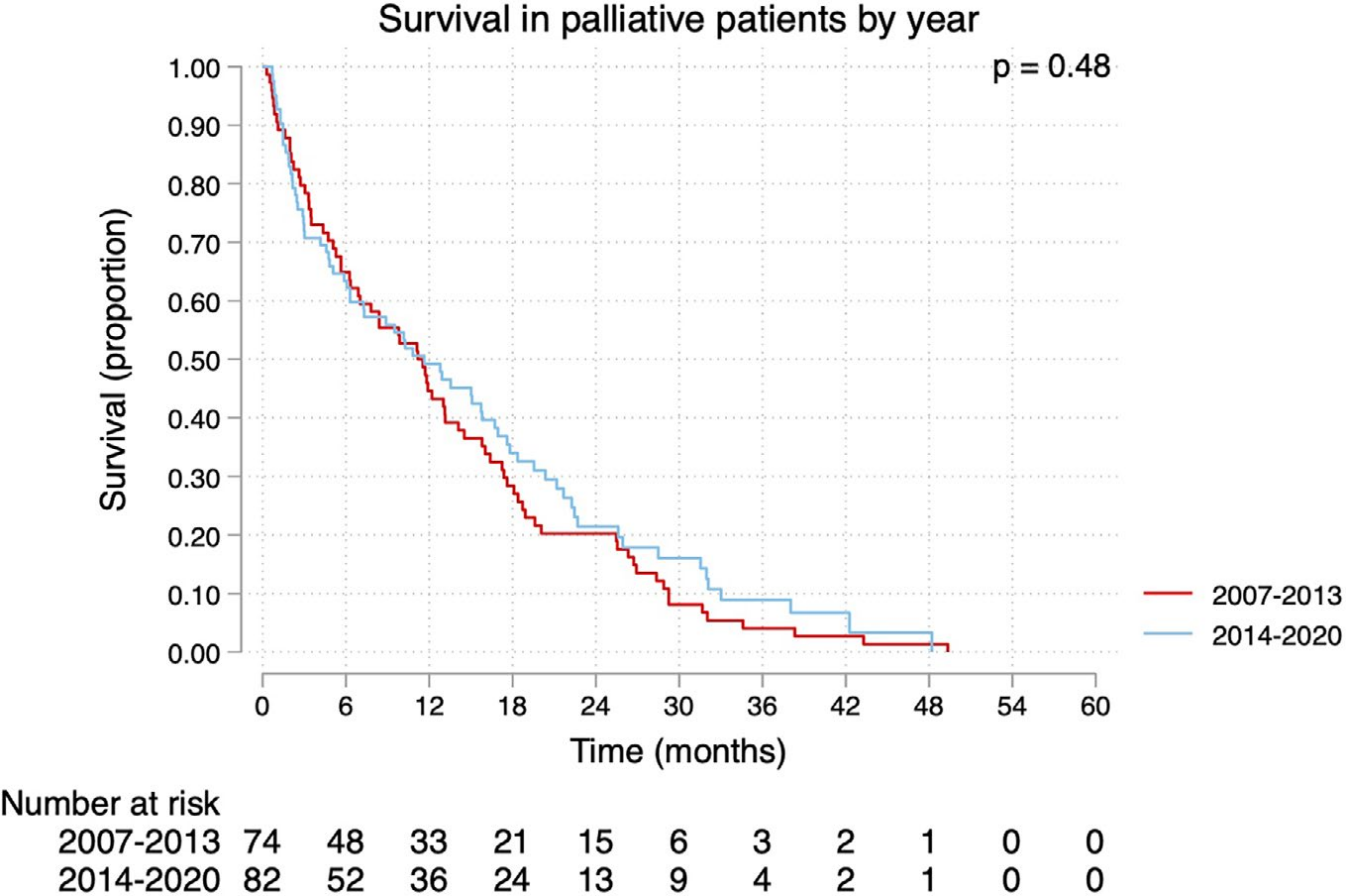
Survival in palliative rectal cancer patients in Region Västerbotten, Sweden, stratified by year of diagnosis.

Table S3 shows stratification by year of diagnosis (2007–2013 and 2014–2020, respectively). In the earlier group, more patients received a diverting stoma compared to the latter group (40/74, 54% vs. 22/82, 27%; *p* = 0.001). They also had a lower incidence of tumour perforation compared to the second group (2/74, 3% vs. 9/82, 11%; *p* = 0.044), and more patients received a diverting stoma with a relative indication in the earlier group compared to the latter group (24/74, 32% vs. 12/82, 15%; *p* < 0.001). Both groups had a similar median survival with 11.6 months (95% CI: 6.3–16.7) in the latter group, compared to 11.2 months (95% CI: 6.9–14.1) in the earlier group (Figure 3).

### Stratification by age

**FIGURE 4:**
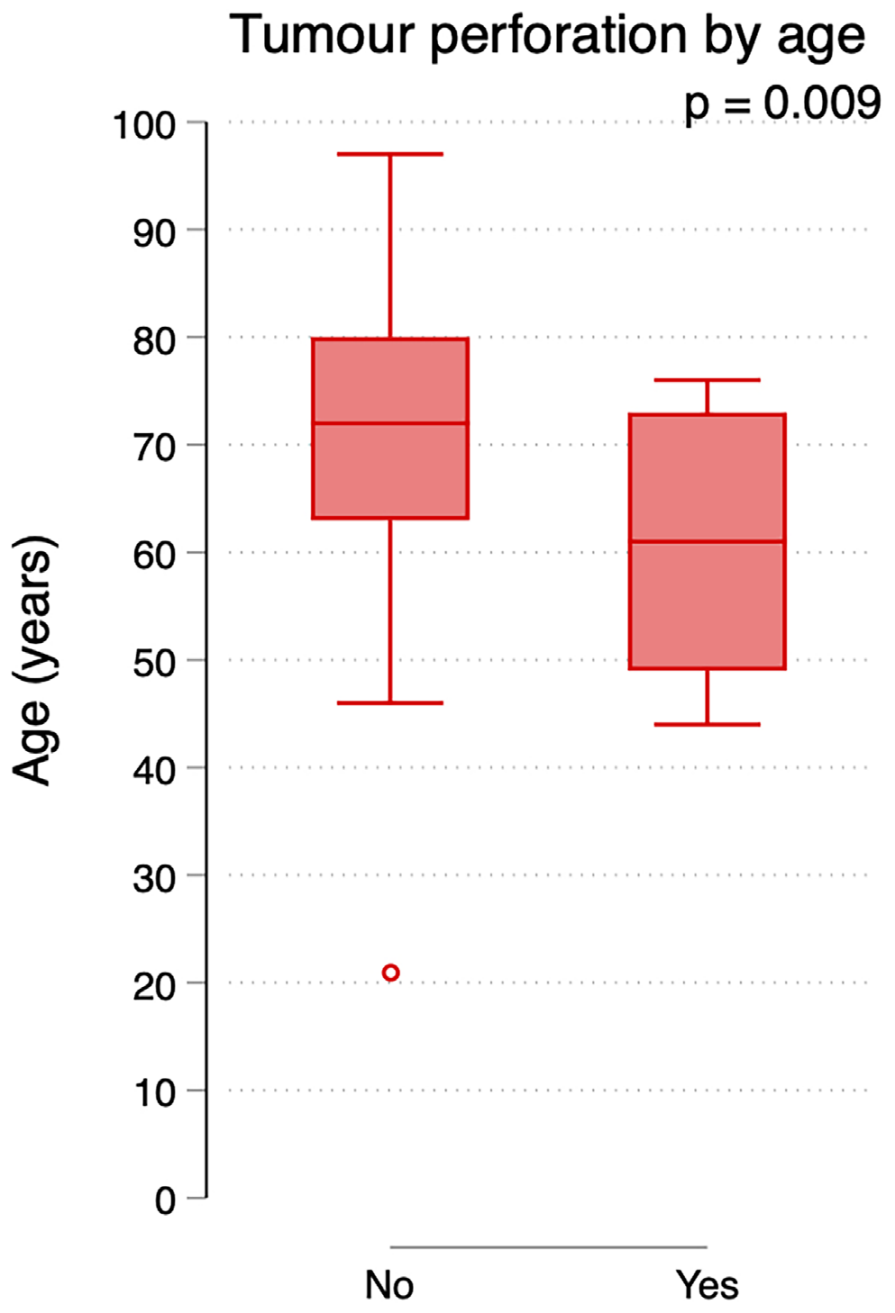
Patient age when diagnosed with rectal cancer, related to occurrence of tumour perforation. Only patients with incurable disease as their indication for palliative management are included.

**FIGURE 5:**
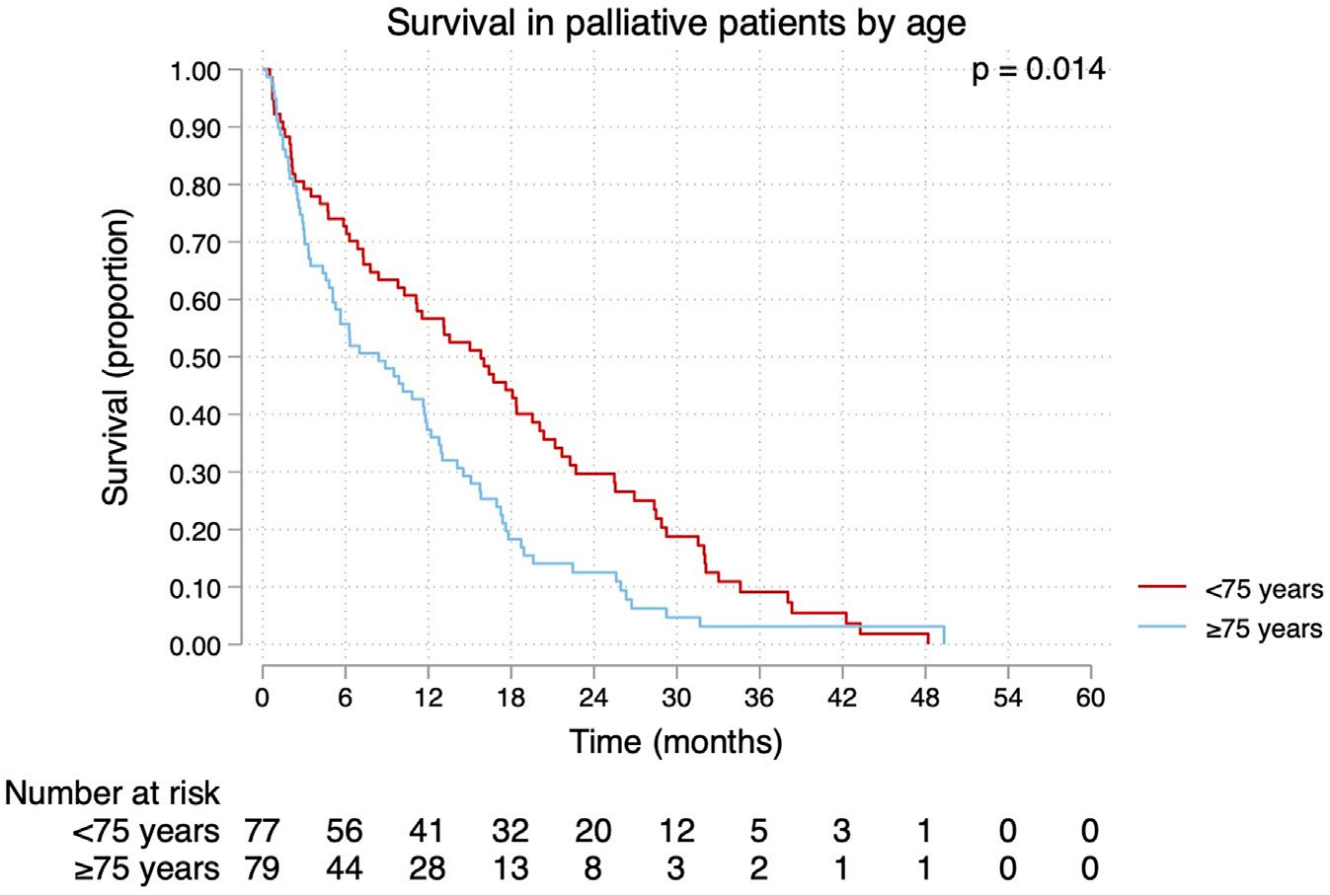
Survival in palliative rectal cancer patients in Region Västerbotten, Sweden, stratified by age.

Table S4 demonstrates stratification by age (<75 years and ≥ 75 years, respectively). Patients aged <75 years suffered tumour perforation more often, compared to those aged ≥75 years (8/77, 10% vs. 3/79, 4%, *p* = 0.11), and younger patients also received a diverting stoma more often compared to older patients (35/77, 45% vs. 19/79, 34%; *p* = 0.19); these differences were not statistically significant. In the subset of patients with incurable disease as the main reason for palliative intent of treatment (Figure 4), younger age was statistically significantly associated with the occurrence of tumour perforation (*p* = 0.009). Survival also differed between the groups, with a median survival of 15.8 months (95% CI: 10.2–19.5) in the younger group compared to 8.4 months (95% CI: 5.1–11.8) in the older group, as shown in Figure 5.

## DISCUSSION AND CONCLUSIONS

The present study demonstrates that, in an unselected group of patients with incurable rectal cancer and the primary tumour left in situ, many patients suffered local complications and required surgical interventions. Of particular importance is that tumour perforation occurred more often in younger patients with a rising incidence over time. Notably, the use of diverting stomas declined to some degree in the latter part of the study period.

One advantage of the present study was its population‐based nature, as it included all patients diagnosed with rectal cancer in a well‐defined geographical region, which mitigates selection bias. However, despite the 14‐year study period, the sample size was limited. Nevertheless, when compared to similar studies [1, 7, 8], it can still be considered relatively large. The long study period encompasses changes in surgical and oncological management and reflects trends in demographics and healthcare resources, thus introducing unavoidable heterogeneity. Another weakness of this study was its retrospective design, with obvious limitations when interpreting patient charts of varying quality. This is particularly relevant when assessing indications for surgery or occurrence and severity of symptoms. For example, the different categories for diverting stoma indications, as well as cancer‐related pain, were increasingly difficult to define in patients nearing the end of their lives, presumably as patient charts may not have been as detailed when patients approached supportive care only.

In the most comparable study by Tan et al., stage IV rectal cancer patients treated with both palliative and curative intent were included, which differed from the present study [1]. However, when excluding the 168 patients who underwent an elective primary tumour resection after systemic therapy was started, thereby making the cohorts more comparable, Tan et al. reported a primary tumour‐related complication rate of 36%, which is similar to our complication rate of 44%. Surgical intervention was performed in 25% of cases with stoma diversion being the most common procedure, approaching the findings from our study. Pain and haematochezia were more frequent in our cohort, perhaps reflecting differences in definitions and healthcare settings. However, the rates of bowel obstruction and perforation, respectively, were similar in both studies. Another study by Nitzkorski et al. included a mix of rectal and colon cancer patients and found that primary tumour‐related symptoms necessitating operative or endoscopic management affected 9.8% of patients who did not undergo resection or colostomy/bypass surgery [7]. A possible explanation to those much lower complication rates might be that rectal tumours are perhaps more likely to cause complications. Another theory is that their cohort only included patients eligible for chemotherapy, while more than half of the patients in our study did not receive any such treatment. This suggests that our cohort included patients with more advanced tumour burden or concurrent disease, making complications more likely to occur. A similar prospective study by Poultsides et al. also including a mix of rectal and colon cancer patients with stage IV disease, concluded that 213 patients (89%) never required any direct symptomatic management for the primary tumour [8]. However, out of these 213 patients, 23% underwent elective tumour resection, thereby effectively removing the risk of tumour‐related bowel obstruction or perforation.

As demonstrated in the present study, patients with incurable rectal cancer can in many cases expect to survive for over a year, especially if diagnosed at a younger age. In this subset of patients, there might be ample time for tumour progression and subsequent perforation, as suggested by the higher rate of perforation found here. Another explanation might be that the tumour biology is more aggressive in younger patients [9]. However, another explanation might be that the higher frequency of genetic mutations found in early onset colorectal cancer compared to those diagnosed later in life [10] might contribute to the increased risk of tumour perforation.

Before introduction of the total mesorectal excision approach to rectal cancer surgery [11], locoregional recurrence was a frequent and debilitating event [12]. Thankfully, refined surgical technique and neoadjuvant therapy have made such scenarios less common [13]. However, the past experiences of patients with locoregional recurrence could serve as a cautionary example for always leaving primary tumours in situ, as these situations share many similarities. Thus, selectively performing prophylactic resections might be beneficial even for palliative patients, despite the lack of a discernible survival advantage. Speculatively, the apparent rise of tumour perforations with time in this study might also be a consequence of less use of diverting stomas, admittedly keeping in mind that most perforations took place either at diagnosis or despite a stoma in place. Moreover, there is also an obvious risk of delaying palliative chemotherapy due to complications from palliative tumour resection [2], making such a decision difficult and, ultimately, tailored to the individual patient. Offering palliative patients a diverting stoma upfront could be an alternative to reduce risk of progression to bowel obstruction, not the least in the light that 40% of patients in the present study ultimately required a stoma; however, it has been demonstrated that diverting stoma surgery in itself is not risk‐free and could lead not only to treatment delay, but also to even severe postoperative complications [14, 15].

The natural course of unresected rectal cancer is an understudied topic. Treatment options are seldom risk‐free, as is the potential progression of the unremoved primary tumour. While oncological treatments keep improving, the surgical strategy needs to be considered in tandem. This study has shown that patients with incurable rectal cancer and an unremoved primary tumour are at risk of local complications often necessitating surgical intervention, and that the risk is dependent on patient characteristics. We also found that despite faecal diversion, tumour perforation can still occur, suggesting a palliative primary tumour resection might have been preferable for some patients. More studies are needed to evaluate whether some patients should be offered palliative resections or not, and how to appropriately select such patients.

## AUTHOR CONTRIBUTIONS

**Gustav Kejving:** Data curation; resources; writing – original draft; writing – review and editing; investigation; conceptualization; visualization; project administration. **Gustav Sandén:** Investigation; writing – original draft; writing – review and editing; formal analysis; data curation. **Ingrid Ljuslinder:** Writing – review and editing; formal analysis. **Jörgen Rutegård:** Formal analysis; writing – review and editing. **Petrus Vinnars:** Writing – review and editing; formal analysis. **Martin Rutegård:** Conceptualization; investigation; funding acquisition; writing – original draft; methodology; validation; visualization; writing – review and editing; software; formal analysis; project administration; data curation; supervision; resources.

## FUNDING INFORMATION

Financial support was provided by the Swedish Cancer Society (23 3056 Fk) and Region Västerbotten (HSN 530–2022).

## CONFLICT OF INTEREST STATEMENT

None to declare.

## ETHICS STATEMENT

The Swedish Ethical Review Authority (dnr 2021–01416 with complementary dnr 2023–05853‐02) approved this study.

## Supporting information


**Table S1.** Comorbidities in the 156 palliative rectal cancer patients Region Västerbotten, Sweden, during 2007–2020.

**Table S2.** Chemotherapy regimens in 156 palliative rectal cancer patients in Region Västerbotten, Sweden, during 2007–2020.

**Table S3.** Outcomes in 156 palliative rectal cancer patients in Region Västerbotten, Sweden, during 2007–2020, stratified by year of diagnosis.

**Table S4.** Outcomes of 156 palliative rectal cancer patients in Region Västerbotten, Sweden, during 2007–2020, stratified by age.

## Data Availability

The data that support the findings of this study are available from the corresponding author upon reasonable request.
