# Supplementary material for: A population‐based study of palliative rectal cancer patients with an unremoved primary tumour: Symptoms, complications and management
Source: Colorectal Dis. 2025 Apr 23;27(4):e70104. doi: 10.1111/codi.70104 (PMC12018725; doi:10.1111/codi.70104)
Supplement: Supplementary file 1 — Table S1. Comorbidities in the 156 palliative rectal cancer patients Region Västerbotten, Sweden, during 2007–2020. [file CODI-27-0-s003.docx]

**Table S1.** Comorbidities in the 156 palliative rectal cancer patients Region Västerbotten, Sweden, during 2007–2020.

| Comorbidities and definitions | N (%) |
| --- | --- |
| Heart disease  Ischaemic and congestive heart disease, cardiomyopathy, pathologies of the electrical conduction system of the heart, or valvular disease | 25 (16.0%) |
| Lung disease  Chronic hypersensitivity pneumonitis, chronic obstructive pulmonary disease, asthma, pulmonary fibrosis, or emphysema | 17 (10.1%) |
| Neurological disease  Multiple sclerosis, developmental disorders, ischaemic brain injury, epilepsy, psychiatric disorders such as schizoaffective syndrome, or severe alcohol abuse | 7 (4.5%) |
| Kidney disease  Membranous nephropathy, or renal failure | 1 (0.6%) |
| Rheumatic disease  Polymyalgia rheumatica | 2 (1.3%) |
| Diabetes mellitus  Type 1 and 2 | 24 (7.1%) |
| Venous thrombotic disease  Deep venous thrombosis, or pulmonary embolism | 3 (1.9%) |
| Dementia  Alzheimer’s disease, Parkinson’s disease, or dementia related to ischaemic events | 24 (15.4%) |
| No comorbidities | 66 (42.3%) |
