# Supplementary material for: A population‐based study of palliative rectal cancer patients with an unremoved primary tumour: Symptoms, complications and management
Source: Colorectal Dis. 2025 Apr 23;27(4):e70104. doi: 10.1111/codi.70104 (PMC12018725; doi:10.1111/codi.70104)
Supplement: Supplementary file 2 — Table S2. Chemotherapy regimens in 156 palliative rectal cancer patients in Region Västerbotten, Sweden, during 2007–2020. [file CODI-27-0-s004.docx]

**Supplementary Table 2.** Chemotherapy regimens in 156 palliative rectal cancer patients in Region Västerbotten, Sweden, during 2007–2020.

|  | Total (N=156) |
| --- | --- |
| Type of palliative chemotherapy |  |
| None | 82 (52.6%) |
| FOLFOX/CAPOX | 31 (19.9%) |
| FOLFOX/CAPOX with EGFR-inhibitor/bevacizumab | 6 (3.8%) |
| FOLFIRI/XELIRI | 12 (7.7%) |
| Fluoropyrimidine monotherapy | 1 (0.6%) |
| Fluorouracil/calciumfolinate (FLV) | 13 (8.3%) |
| Fluorouracil with EGFR-inhibitor/bevacizumab | 8 (5.1%) |
| FLV/vectibix | 2 (1.3%) |

FOLFOX=Folinic acid, fluorouracil and oxaliplatin; CAPOX=Capecitabine and oxaliplatin; EGFR=Epidermal growth factor receptor; FOLFIRI=Folinic acid, fluorouracil and irinotecan; XELIRI=Capecitabine and irinotecan; FLV=Fluorouracil and folinic acid.
