# Supplementary material for: A population‐based study of palliative rectal cancer patients with an unremoved primary tumour: Symptoms, complications and management
Source: Colorectal Dis. 2025 Apr 23;27(4):e70104. doi: 10.1111/codi.70104 (PMC12018725; doi:10.1111/codi.70104)
Supplement: Supplementary file 3 — Table S3. Outcomes in 156 palliative rectal cancer patients in Region Västerbotten, Sweden, during 2007–2020, stratified by year of diagnosis. [file CODI-27-0-s001.docx]

**Table S3.** Outcomes in 156 palliative rectal cancer patients in Region Västerbotten, Sweden, during 2007–2020, stratified by year of diagnosis.

|  | 2007–2013 (N=74) | 2014–2020 (N=82) | p-value |
| --- | --- | --- | --- |
| Severe haematochezia |  |  | 0.40 |
| No | 68 (91.9%) | 72 (87.8%) |  |
| Yes | 6 (8.1%) | 10 (12.2%) |  |
| Pain |  |  | 0.28 |
| No pain reported | 55 (74.3%) | 55 (67.1%) |  |
| Diffuse extra-abdominal pain | 2 (2.7%) | 7 (8.5%) |  |
| Diffuse abdominal pain | 8 (10.8%) | 13 (15.9%) |  |
| Localised pain due to primary tumour | 9 (12.2%) | 7 (8.5%) |  |
| Tumour perforation |  |  | 0.044 |
| No | 72 (97.3%) | 73 (89.0%) |  |
| Yes | 2 (2.7%) | 9 (11.0%) |  |
| Degree of bowel obstruction |  |  | 0.17 |
| None | 57 (77.0%) | 63 (76.8%) |  |
| Partial | 3 (4.1%) | 9 (11.0%) |  |
| Complete | 14 (18.9%) | 10 (12.2%) |  |
| Type of stoma |  |  | 0.002 |
| No stoma | 34 (45.9%) | 60 (73.2%) |  |
| Ileostomy | 9 (12.2%) | 3 (3.7%) |  |
| Colostomy | 27 (36.5%) | 19 (23.2%) |  |
| Other | 4 (5.4%) | 0 (0.0%) |  |
| Reason for stoma |  |  | <0.001 |
| No stoma | 34 (45.9%) | 60 (73.2%) |  |
| Obstruction/perforation: absolute indication | 7 (9.5%) | 9 (11.0%) |  |
| Obstruction/pain/bleeding: relative indication | 24 (32.4%) | 12 (14.6%) |  |
| Prophylactic | 9 (12.2%) | 1 (1.2%) |  |
| Surgical intervention |  |  | 0.017 |
| No | 31 (41.9%) | 50 (61.0%) |  |
| Yes | 43 (58.1%) | 32 (39.0%) |  |
| Postoperative complications (Clavien-Dindo grade) within 90 days |  |  | 0.164 |
| 0–I | 31 (73.8%) | 20 (62.5%) |  |
| II | 3 (7.1%) | 6 (18.8%) |  |
| IIIa | 3 (7.1%) | 1 (3.1%) |  |
| IIIb | 1 (2.4%) | 4 (12.5%) |  |
| IV | 0 (0%) | 0 (0%) |  |
| V | 4 (9.5%) | 1 (3.1%) |  |
