# Supplementary material for: A population‐based study of palliative rectal cancer patients with an unremoved primary tumour: Symptoms, complications and management
Source: Colorectal Dis. 2025 Apr 23;27(4):e70104. doi: 10.1111/codi.70104 (PMC12018725; doi:10.1111/codi.70104)
Supplement: Supplementary file 4 — Table S4. Outcomes of 156 palliative rectal cancer patients in Region Västerbotten, Sweden, during 2007–2020, stratified by age. [file CODI-27-0-s002.docx]

**Table S4.** Outcomes of 156 palliative rectal cancer patients in Region Västerbotten, Sweden, during 2007–2020, stratified by age.

|  | <75 years (N=77) | ≥75 years (N=79) | p-value |
| --- | --- | --- | --- |
| Severe haematochezia |  |  | 0.32 |
| No | 71 (92.2%) | 69 (87.3%) |  |
| Yes | 6 (7.8%) | 10 (12.7%) |  |
| Pain category |  |  | 0.25 |
| No pain reported | 51 (66.2%) | 59 (74.7%) |  |
| Diffuse extra-abdominal pain | 3 (3.9%) | 6 (7.6%) |  |
| Diffuse abdominal pain | 14 (18.2%) | 7 (8.9%) |  |
| Localised pain due to primary tumour | 9 (11.7%) | 7 (8.9%) |  |
| Tumour perforation |  |  | 0.11 |
| No | 69 (89.6%) | 76 (96.2%) |  |
| Yes | 8 (10.4%) | 3 (3.8%) |  |
| Degree of bowel obstruction |  |  | 0.27 |
| None | 55 (71.4%) | 65 (82.3%) |  |
| Partial | 7 (9.1%) | 5 (6.3%) |  |
| Complete | 15 (19.5%) | 9 (11.4%) |  |
| Type of stoma |  |  | 0.44 |
| No stoma | 42 (54.5%) | 52 (65.8%) |  |
| Ileostomy | 8 (10.4%) | 4 (5.1%) |  |
| Colostomy | 25 (32.5%) | 21 (26.6%) |  |
| Other | 2 (2.6%) | 2 (2.5%) |  |
| Reason for stoma |  |  | 0.031 |
| No stoma | 42 (54.5%) | 52 (65.8%) |  |
| Obstruction/perforation: absolute indication | 9 (11.7%) | 7 (8.9%) |  |
| Obstruction/pain/bleeding: relative indication | 24 (31.2%) | 12 (15.2%) |  |
| Prophylactic | 2 (2.6%) | 8 (10.1%) |  |
| Surgical intervention |  |  | 0.025 |
| No | 33 (42.9%) | 48 (60.8%) |  |
| Yes | 44 (57.1%) | 31 (39.2%) |  |
| Postoperative complications (Clavien-Dindo grade) within 90 days |  |  | 0.342 |
| 0–I | 27 (61.4%) | 24 (80.0%) |  |
| II | 7 (15.9%) | 2 (6.7%) |  |
| IIIa | 4 (9.1%) | 0 (0.0%) |  |
| IIIb | 3 (6.8%) | 2 (6.7%) |  |
| IV | 0 (0%) | 0 (0%) |  |
| V | 3 (6.8%) | 2 (6.7%) |  |
